# Neutrophils Lose the Capacity to Suppress T Cell Proliferation Upon Migration Towards Inflamed Joints in Juvenile Idiopathic Arthritis

**DOI:** 10.3389/fimmu.2021.795260

**Published:** 2022-01-13

**Authors:** Sabine Arve-Butler, Anki Mossberg, Tobias Schmidt, Charlotte Welinder, Hong Yan, Elisabet Berthold, Petra Król, Robin Kahn

**Affiliations:** ^1^ Department of Rheumatology, Clinical Sciences Lund, Lund University, Lund, Sweden; ^2^ Wallenberg Center for Molecular Medicine, Lund University, Lund, Sweden; ^3^ Department of Pediatrics, Clinical Sciences Lund, Lund University, Lund, Sweden; ^4^ Department of Clinical Sciences, Division of Oncology, Lund University, Lund, Sweden; ^5^ Swedish National Infrastructure for Biological Mass Spectrometry, Biological Mass Spectrometry (BioMS), Lund, Sweden

**Keywords:** neutrophil, T cell, juvenile idiopathic arthritis, suppression, reactive oxygen species

## Abstract

Neutrophils are highly abundant in synovial fluid of rheumatic inflamed joints. In oligoarticular juvenile idiopathic arthritis (JIA), synovial fluid neutrophils have impaired effector functions and altered phenotype. We hypothesized that these alterations might impact the immunoregulatory interplay between neutrophils and T cells. In this study we analyzed the suppressive effect of neutrophils, isolated from blood and synovial fluid of oligoarticular JIA patients, on CD4^+^ T cells activated by CD3/CD28 stimulation. JIA blood neutrophils suppressed T cell proliferation but synovial fluid neutrophils from several patients did not. The loss of T cell suppression was replicated in an *in vitro* transmigration assay, where healthy control neutrophils migrated into synovial fluid through transwell inserts with endothelial cells and synoviocytes. Non-migrated neutrophils suppressed proliferation of activated CD4^+^ T cells, but migrated neutrophils had no suppressive effect. Neutrophil suppression of T cells was partly dependent on reactive oxygen species (ROS), demonstrated by impaired suppression in presence of catalase. Migrated neutrophils had reduced ROS production compared to non-migrated neutrophils. A proteomic analysis of transwell-migrated neutrophils identified alterations in proteins related to neutrophil ROS production and degranulation, and biological processes involving protein transport, cell-cell contact and inflammation. In conclusion, neutrophils in synovial fluid of children with JIA have impaired capacity to suppress activated T cells, which may be due to reduced oxidative burst and alterations in proteins related to cell-cell contact and inflammation. The lack of T cell suppression by neutrophils in synovial fluid may contribute to local inflammation and autoimmune reactions in the JIA joint.

## Introduction

Swollen and painful joints are characteristics of many autoimmune rheumatic diseases. In several of these diseases, neutrophils in synovial fluid of inflamed joints have been found to have altered phenotype and behavior, driving local inflammation (1–5). Neutrophils are not only pro-inflammatory mediators but also immunoregulators, where one of their immunoregulatory effects is the capacity to suppress T cell proliferation and cytokine production (6–9). Neutrophil suppression of T cells has been described in both health and disease, by mechanisms involving reactive oxygen species (ROS), arginase-1, direct cell-cell contact and anti-inflammatory cytokines (6, 10–12).

Juvenile idiopathic arthritis (JIA) is the most common rheumatic joint disease in the pediatric population. JIA includes several different subtypes covering a spectrum of diseases where the most prevalent subtype in the western world is oligoarticular JIA, representing 30-60% of all JIA cases (13–15). Children with oligoarticular JIA present with persistent, unexplained and often asymmetric arthritis in one to four joints before the age of sixteen, often associated with anti-nuclear autoantibodies (13). We and others have shown that neutrophils in synovial fluid from children with oligoarticular JIA are activated and express atypical neutrophil surface markers (2, 5). JIA synovial fluid neutrophils also have impaired phagocytosis and trend towards impaired oxidative burst compared to circulating neutrophils, related to the phenotype shift (2).

Activated CD4^+^ memory T cells are enriched in synovial fluid of inflamed joints in children with oligoarticular JIA (16, 17) and T cells can be found in lymphoid aggregates in the synovial tissue (18). Synovial T cells in JIA are primarily of Th1 or Th17 lineages (16, 19). T cell activation and impaired suppression of overactive T cells appears to be important in JIA pathogenesis as JIA synovial fluid effector T cells are resistant to suppression by regulatory T cells (Tregs) (17), and peripheral blood Tregs have impaired suppressive activity during disease flares (20). Additionally, the immunomodulatory drug abatacept, preventing T cell activation by blocking the co-stimulatory molecules CD80 and CD86, has proven successful in the treatment of JIA (21).

However, no studies have investigated the role of T cell suppression by neutrophils in JIA, and only very few studies have examined T cell suppression by myeloid cells in synovial fluid in other rheumatic diseases. In these studies, suppressive myeloid cells, primarily of granulocytic origin, have been found in synovial fluid from patients with rheumatoid arthritis (RA) (22, 23) and mouse models of arthritis (23, 24). None of these studies compared the suppressive capacity of synovial cells with circulating neutrophils. To the best of our knowledge, the effect of neutrophil migration on T cell suppressive capacity has never been investigated.

This study investigates the interplay between neutrophils and T cells in oligoarticular JIA, and the effect of tissue migration towards the inflamed joint on neutrophil suppressive capacity. We hypothesize that neutrophils in synovial fluid might be less efficient immunoregulators, thus contributing to the sustained inflammation in the joint.

## Methods

### Patient Material and Healthy Controls

16 children with JIA, fulfilling the International League of Associations for Rheumatology (ILAR) criteria for oligoarticular JIA, undergoing therapeutic synovial fluid aspiration were included in the study. None of the patients had been treated with anti-rheumatic drugs, such as steroids or conventional or biologic disease-modifying anti-rheumatic drugs (DMARDs), for at least six months prior to study inclusion with the exception of non-steroid anti-inflammatory drugs (NSAIDs). Neutrophils were studied in blood (n=9) and synovial fluid (n=11) in patients 1-11 (**Table 1**). Cell-free synovial fluid from patients 9-16 were used as stimuli for *in vitro* assays. Clinical characteristics and samples used are summarized in **Table 1**. In the samples where patient neutrophils were studied, blood- and synovial fluid leukocyte counts were analyzed on an XN-350 differential hematology analyzer (Sysmex Corporation), **Supplementary Table 1**. Healthy adult controls participated with blood samples.

**Table 1:** Clinical characterization of the patient cohort.

	Patient sample	Assay performed	Age (years)	Sex	Disease duration (months)	ANA
**1**	Blood, SF	*Ex vivo* ^1^	11	F	64	Neg
**2**	SF	*Ex vivo* ^1^	14	F	9	Neg
**3**	Blood, SF	*Ex vivo* ^1^	3	F	12	Pos
**4**	SF	*Ex vivo* ^1^	5	M	1	Pos
**5**	Blood, SF	*Ex vivo* ^1^	3	F	<1	Pos
**6**	Blood, SF	*Ex vivo* ^1^	11,5	F	<1	Pos
**7**	Blood, SF	*Ex vivo* ^1^	3,5	F	<1	Pos
**8**	Blood, SF	*Ex vivo* ^1^	11	F	105	Pos
**9**	Blood SF	*Ex vivo* ^1^, *in vitro* ^2^	15	M	<1	Neg
**10**	Blood, SF	*Ex vivo* ^1^, *in vitro* ^2^	11,5	M	103	Pos
**11**	Blood, SF	*Ex vivo* ^1^, *in vitro* ^2^	8,5	F	68	Pos
**12**	SF	*In vitro* ^2^	17	F	144	Neg
**13**	SF	*In vitro* ^2^	15	F	<1	Pos
**14**	SF	*In vitro* ^2^	16,5	F	48	Pos
**15**	SF	*In vitro* ^2^	15,5	F	<1	Neg
**16**	SF	*In vitro* ^2^	15	M	132	Pos

ANA, antinuclear antibodies; SF, synovial fluid; F, female; M, male; neg, negative; pos, positive.^1^Patient neutrophils used in T cell suppression and neutrophil surface marker analyses. ^2^Cell-free synovial fluid used in transmigration assay.

The study was approved by the Regional Ethical Review Board for southern Sweden (LU2016/128). Informed consent was obtained from the healthy control, patient, and/or the patients’ guardians.

### Neutrophil and CD4^+^ T Cell Isolation

Heparinized whole blood was applied to a Lymphoprep (Axis-Shield) density gradient and centrifuged as per manufacturer’s instructions. Peripheral blood mononuclear cells (PBMCs) were collected and washed with PBS. CD4^+^ T cells were isolated from PBMCs using EasySep™ human CD4^+^ T cell isolation kit (Stemcell). Neutrophils were isolated from the red blood cell pellet by sedimentation of red blood cells in saline with 1.5% dextran T500 (Pharmacosmos). The neutrophil rich supernatant was collected and remaining red blood cells lysed with sterile H_2_O. Healthy donor T cells and neutrophils were obtained from the same blood sample in each experiment, however, several healthy donors were included in the study.

Cells in synovial fluid were pelleted at 400 g, 10 min, and the cell-free synovial fluid collected and stored at -80°C until further experiments. Synovial fluid cells were washed, resuspended in PBS with 0.5% BSA, and separated by Lymphoprep (Axis-Shield) density gradient. Cells in PBMC and granulocyte fractions were washed and analyzed by an XN-350 differential hematology analyzer (Sysmex Corporation). Neutrophils from the granulocyte fraction were used for T cell proliferation experiments if the purity was >95%. Presence of low-density neutrophils in blood and synovial fluid were in median 0,49 (IQR 0,39-0,88) and 1,3 (IQR 1,06-4,04) % of the total neutrophil count respectively.

### T Cell Proliferation Assay

CD4^+^ T cells were labelled with 2 μM CellTrace Violet (Invitrogen, Thermo Fisher Scientific). CD4^+^ T cells and neutrophils were cultured at a 1:1 ratio, 50 000 cells of each cell type, in a 96-well culture plate (Eppendorf) coated with anti-CD3 (clone OKT3, Invitrogen, Thermo Fisher Scientific, 1:500) and anti-CD28 (clone CD28.2, Invitrogen, Thermo Fisher Scientific, 1:1000), in total volume 200 μl RPMI supplemented with 10% fetal calf serum and 2 mM L-glutamine. When indicated, 100 μg/ml catalase (from bovine liver, Sigma) was added to the culture medium. After 3 days of culture at 37°C (5% CO_2_) T cell proliferation was analyzed on a Cytoflex flow cytometer (Beckman Coulter).

Levels of IFNγ in culture supernatants were quantified using Human IFN-Gamma Quantikine ELISA (R&D Systems) according to manufacturer’s instructions. The T cell culture supernatants were collected after 3 days of culture and stored at -20°C until analysis.

### Neutrophil Surface Markers

Neutrophils were stained for 20 min at RT with an antibody panel containing CD66b-FITC 1:20, CD10-PE-Cy7 1:20, CD11b-AlexaFluor 700 1:66, CD16-APC-H7 1:66, CD14-BrilliantViolet 421 1:66, Siglec8-BrilliantViolet 510 1:66 (all from BD) and CD62L-APC 1:66 (Biolegend). Neutrophils were washed and analyzed on a Cytoflex flow cytometer (Beckman Coulter).

### Transmigration Assay

Primary human knee synoviocytes (adult male, Cell Applications) were cultured on the undersides of upside-down transwell inserts with 5 μm pore size (Corning) in synoviocyte growth medium (Cell Applications). After adhesion and growth of the synoviocytes the inserts were turned and placed in wells, HMEC endothelial cells (ATCC) were added to the insert insides and cultured in MCDB 131 medium (Gibco), supplemented with 10% fetal bovine serum, 2 mM L-glutamine, 10 ng/ml hEGF, non-essential amino acids, sodium pyruvate and PenStrep, until confluency of both cell types, assessed by light microscopy of wells seeded with the same cell numbers as the inserts.

Inserts were placed in an ultra-low attachment 24-well cell culture plate in wells with 400 μl MCDB 131 media with 10% synovial fluid. Healthy donor neutrophils, 5x10^5^ in 100 μl MCDB 131, were added to the transwell inserts and incubated for 1 h at 37°C. As migration controls, neutrophils were incubated in wells, without inserts, containing the same synovial fluids or normal serum of the neutrophil donor. After incubation, inserts were removed and neutrophils in the wells were collected to new tubes for further analysis.

### Neutrophil ROS Production

Neutrophils, 5x10^4^ in 100 μl RPMI, were stimulated with 20 nM phorbol-myristate-acetate (PMA) for a total of 20 min at 37°C. After 10 min, 20 μl DHR-123 reagent (from phagoburst kit, BD) was quickly added to each tube followed by another 10 min of incubation at 37°C. Reaction was stopped by placing tubes on ice and a wash with cold PBS before analysis in a Cytoflex flow cytometer (Beckman Coulter).

### Neutrophil Proteomic Analysis

The transmigration assay was performed on healthy control neutrophils allowed to incubate in or migrate towards synovial fluid from three patients. All conditions were performed in triplicates. To purify neutrophils from possible contaminating synoviocytes or HMEC cells, collected neutrophils were labelled with 0.5 μg biotin-anti-CD66b (clone G10F5, Biolegend) and captured on magnetic M280 Streptavidin Dynabeads™ (Invitrogen, Thermo Fisher Scientific). Isolated neutrophils, 7.5x10^4^, were washed twice with PBS before lysis in RIPA (Thermo Scientific) with cOmplete protease inhibitor cocktail (Roche). Proteins were precipitated in 90% EtOH over night at -20°C. Proteins were pelleted, resuspended in PBS and concentration determined by Pierce™ BCA Protein Assay kit (Thermo Fisher).

Proteins were prepared for liquid chromatography mass spectrometry (LC-MS) by reduction in 5 μM dithiothreitol (DTT), alkylation by 10 μM iodoacetamide (IAA) followed by trypsination at trypsin:protein ratio 1:50 o/n at 37°C. Peptide desalting was performed in C18 spin columns. Peptides were dried by SpeedVac, resuspended in 2% acetonitrile (ACN) and 0.1% trifluoroacetic acid (TFA) to 0.5 μg/μl.

Peptides were analyzed by LC-MS on a fusion tribrid mass spectrometer (Thermo Fisher Scientific). One μg of peptides was injected into the LC-MS and concentrated on an Acclaim PepMap RSLC column (75 μm x 25 cm, nanoViper, C18, 2 μm, 100 Å) at 45°C and flow rate 300 nl/min. Peptides were eluted by a nonlinear gradient of 3% to 90% ACN containing 0.1% formic acid for 120 min. The Orbitrap Fusion was operated in the positive data-dependent acquisition (DDA) mode. Full MS survey scans from m/z 350-1350 with a resolution of 120,000 were performed in the Orbitrap detector.

Raw data was processed by Proteome Discoverer version 2.2 (Thermo Fisher Scientific). Peptides were identified using SEQUEST HT against UniProtKB human database (release 2019-01-24). Protein abundances were normalized against total amount of peptides per sample. Data was filtered to include only proteins found in at least two of three triplicates per condition, in at least two of three synovial fluids. Immunoglobulins were removed from protein list due to probable contamination from the neutrophil purification process. Abundances for all proteins were calculated per synovial fluid and condition as triplicate averages. Total migration and incubation average abundances across all synovial fluids were used for fold change analysis. Proteins were considered significantly altered if the average abundance in migrated compared to incubated neutrophils had an absolute Log2 fold change of >0.585 and a p-value of <0.05 in paired t-test per synovial fluid. Enrichment analysis of the significantly altered proteins was performed using ToppGene (https://toppgene.cchmc.org/). Biological processes containing ≥5 significantly altered proteins were studied. If several biological processes from the same gene ontology hierarchical tree were significantly enriched only the most distal was considered.

### Statistics

Paired samples were analyzed by paired *t*-test or Wilcoxon signed-rank test and independent samples by Mann-Whitney *U*-test. Proteomic data was processed and analyzed as described in the previous section. Data analysis was performed in GraphPad Prism 9 and Microsoft Excel.

## Results

### Neutrophils From JIA Blood, but Not Synovial Fluid, Can Suppress T Cell Proliferation

**Figure 1:**
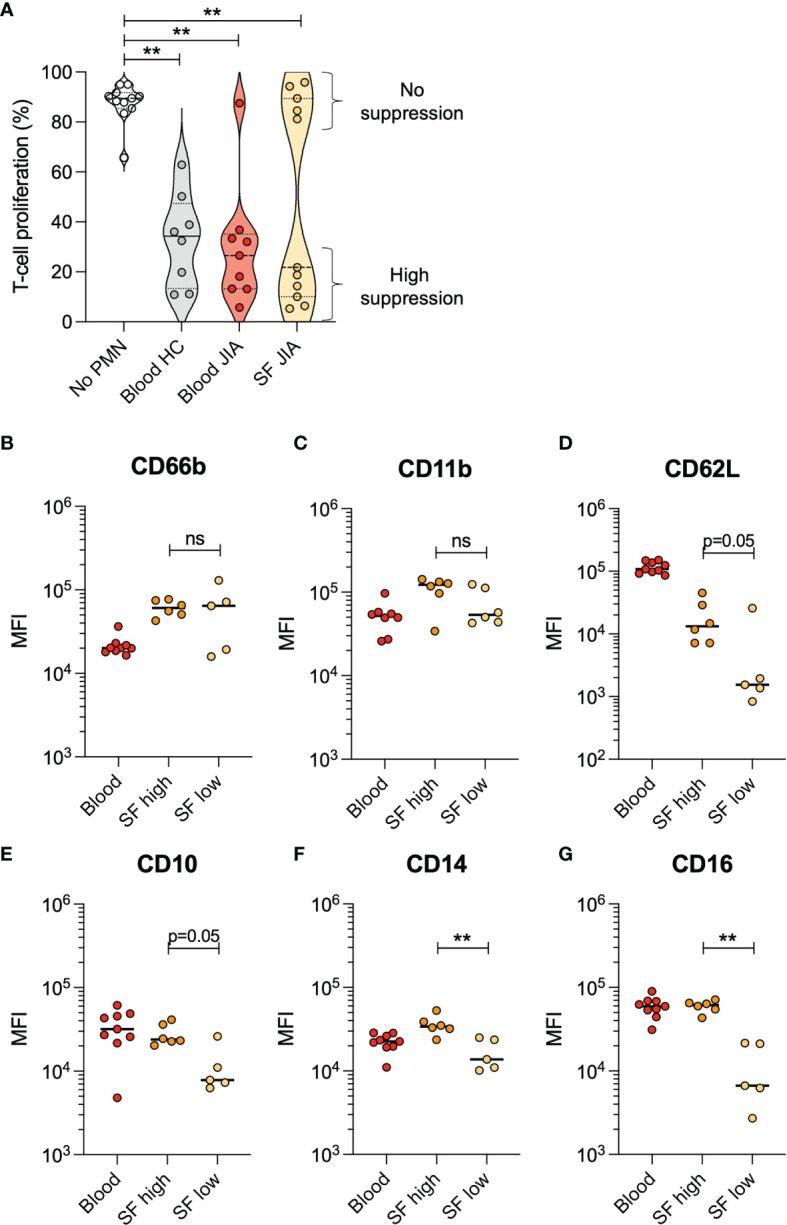
Neutrophils from JIA synovial fluid either have high or no suppressive effect on T cell proliferation. **(A)** Proportion of CD4^+^ T cells in proliferative phase after activation with CD3 and CD28 stimulation, with or without presence of neutrophils (PMN) from healthy control (HC) blood, JIA blood, or JIA synovial fluid (SF). The HC neutrophils and T cells were obtained from a single donor within each experiment. Lines at median and quartiles, **p < 0.01 Wilcoxon signed-rank test. **(B–G)** Surface markers on neutrophils from JIA blood and SF, presented as median fluorescence intensity (MFI). SF neutrophils are categorized based on their suppressive effect on T cell proliferation. Line at median, **p < 0.01 Mann-Whitney U-test. ns, not significant.

To investigate the effect on T cell proliferation by synovial fluid neutrophils from arthritic joints, neutrophils were isolated from oligoarticular JIA blood (n=9) and synovial fluid (n=11) and co-cultured with activated CD4^+^ T cells from a healthy donor. Blood neutrophils from all patients but one suppressed T cell proliferation to a similar extent as blood neutrophils from healthy controls (**Figure 1A**). Interestingly, synovial fluid neutrophils clustered into two groups, where synovial neutrophils from five patients had no suppressive effect on T cell proliferation and synovial neutrophils from six patients had a suppressive effect similar to blood neutrophils (**Figure 1A**).

### Non-Suppressive and Suppressive Synovial Neutrophils Have Different Phenotypes

Non-suppressive synovial fluid neutrophils had low levels of several surface markers compared to suppressive neutrophils. Levels of CD14 and CD16 were significantly lower in non-suppressive neutrophils, and there was a trend (p=0.05) towards lower levels in CD62L and CD10 as well (**Figures 1B–G**). These results are supported by the literature where non-suppressive neutrophils are described as CD10-negative (25), and suppressive neutrophils described as CD16^bright^ (11).

### Migration Over an Artificial Synovial Membrane

**Figure 2:**
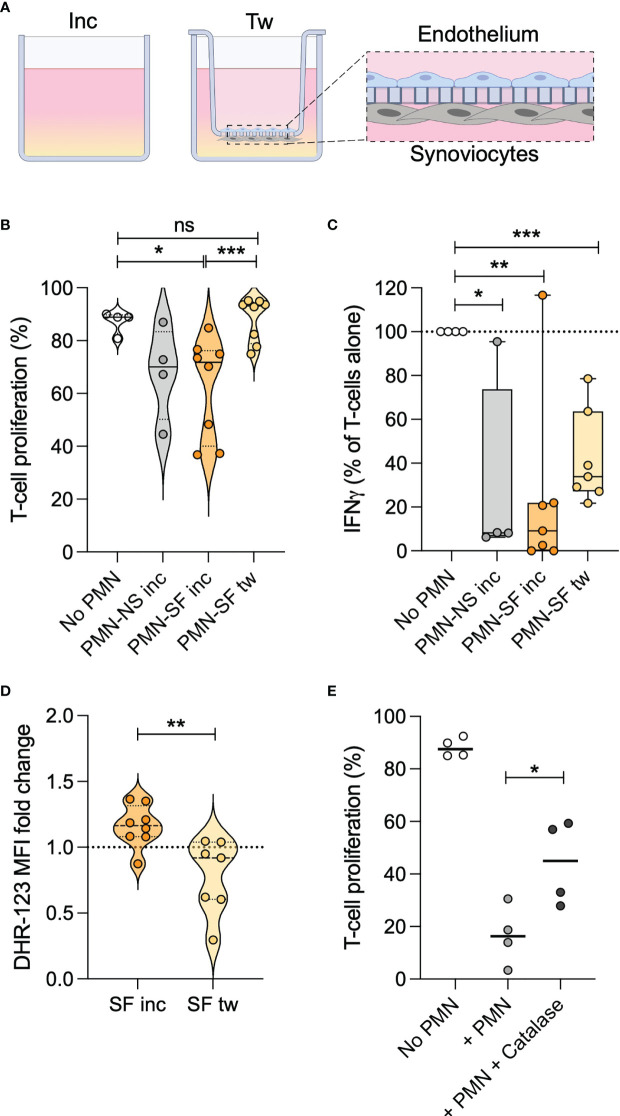
Transmigration causes neutrophils to lose T cell suppressive capacity. **(A)** Schematic of the transmigration system, with transwell inserts covered in endothelial cells and knee synoviocytes. Wells contain 10% synovial fluid (SF), or normal serum (NS) in control wells. Neutrophils are added to transwell inserts or control wells. After 1h, inserts were removed, and neutrophils collected from wells. Created with BioRender.com. **(B)** Proportion of activated CD4^+^ T cells in proliferative phase, with or without neutrophils stimulated with SF or NS in control wells (inc) or migrated through transwell inserts (tw). **(C)** Relative amount of IFNγ in T cell culture supernatants after 3 days, normalized towards levels in supernatants of activated T cells without neutrophils. **(D)** ROS production, quantified by DHR-123, in PMA-stimulated neutrophils after 1 h incubation in or transwell migration towards SF, compared to incubation in NS. **(E)** Proportion of activated CD4^+^ T cells in proliferative phase, with or without neutrophils and 100 µg/ml catalase. In each experiment, neutrophils and T cells were obtained from a single healthy donor. Line at median, *p < 0.05, **p < 0.01, ***p < 0.001 paired t-test. ns, not significant.

To study neutrophil migration from the bloodstream into an inflamed joint, we created a model of a synovial membrane. This model migration system consisted of transwell inserts covered with endothelial cells and synoviocytes, placed in wells with medium containing synovial fluid (**Figure 2A**), where healthy donor neutrophils were allowed to migrate through the inserts towards the synovial fluid. The experiment was performed using individual synovial fluids from patients 9-15. As a migration control, neutrophils were incubated with the same JIA synovial fluids, or normal serum from the healthy donor, in wells without transwell inserts.

To verify that neutrophils had actively migrated, we studied the phenotype of migrated or incubated neutrophils. The surface marker CD62L was increased in neutrophils exposed to synovial fluid compared to normal serum, and significantly reduced after migration indicating an active migration process. None of the other markers studied were altered in migrated- compared to non-migrated neutrophils (**Supplementary Figure 1**).

### Neutrophils Lose Their T Cell Suppressive Capacity Upon *In Vitro* Transmigration

As neutrophil suppression of T cell proliferation was completely absent in synovial fluid neutrophils in several JIA patients studied, we wanted to investigate if transmigration towards synovial fluid in our model would affect the capacity of healthy neutrophils to suppress T cells.

Like the non-suppressive JIA neutrophils in synovial fluid, healthy donor neutrophils completely lost their suppressive effect on T cell proliferation upon migration towards all synovial fluids (**Figure 2B**). In contrast, neutrophils incubated in the same synovial fluids without migration retained their inhibitory effect. Activated T cells produce large amounts of IFNγ, which was also suppressed by the presence of neutrophils (**Figure 2C**). Migrated neutrophils seemed to have less inhibitory effect on IFNγ production compared with non-migrated neutrophils (**Figure 2C**), in line with the diminished suppressive effect on T cell proliferation.

Together, the results demonstrate that transmigration mediates a loss of neutrophil suppression of CD4^+^ T cell proliferation, and impaired inhibition of T cell IFNγ production. The impairment of suppressive capacity is associated with the migration process, rather than exposure to synovial fluid.

### Transmigrated Neutrophils Have Impaired Oxidative Burst

We have previously shown that neutrophils in JIA synovial fluid trend towards reduced oxidative burst compared to neutrophils in the blood (2), and we therefore sought to investigate if migration in our system would affect neutrophil ROS production. When exposed to PMA, neutrophils which had migrated towards synovial fluid had impaired ROS production compared to neutrophils incubated in the same synovial fluid (**Figure 2D**). The impairment was consistent in all samples, and transmigrated neutrophils had a ROS production of 27-83% of that of non-migrated neutrophils incubated in the same synovial fluid. Thus, we concluded that our *in vitro* migration system, at least partly, resembles the *in vivo* process of neutrophil migration into joints.

Oxidative burst is described to be essential for neutrophil suppression of T cell proliferation (9, 26–28). Presence of catalase in the T cell proliferation assay could partly rescue the T cell proliferation suppressed by neutrophils (**Figure 2E**). Therefore, the decrease in ROS following neutrophil migration is likely to be involved in, but probably doesn’t fully explain, the loss of T cell suppressive capacity.

### Neutrophil Proteomic Alterations Upon Transmigration

We performed a proteomic analysis of neutrophils incubated in or migrated towards synovial fluid from patients 9, 14 and 16 in the transwell system, to further explore possible mechanisms for the loss of T cell suppressive capacity following transmigration towards synovial fluid.

**Figure 3:**
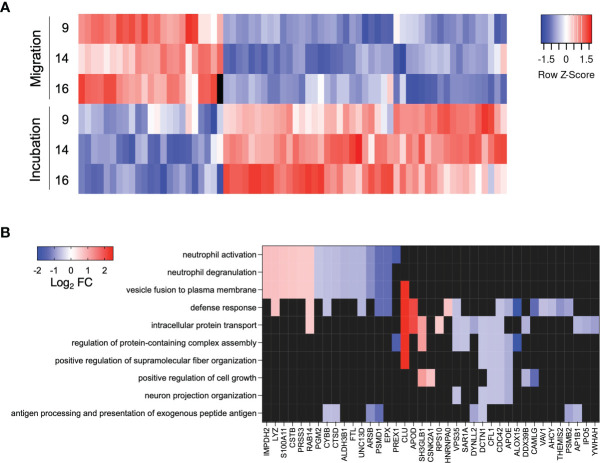
Significantly altered proteins and enriched biological processes. **(A)** Heat map of abundance of significantly altered proteins. Colors indicate protein abundance z-scores. **(B)** Heat map of significantly altered proteins included in the top ten enriched GO biological processes. Colors indicate Log2 fold change of the protein abundance in migrated neutrophils compared to neutrophils incubated in the same synovial fluid.

We identified 68 significantly altered proteins (**Supplementary Table 2**), most of which were less abundant in migrated- compared to incubated neutrophils (**Figure 3A**). The protein with largest increase in abundance after migration was clusterin (CLU), fold change 5.26 (**Supplemental Table 1**, **Figure 3B**), a secreted protein with multiple functions involving cell survival, adhesion and immunological processes (29, 30). The protein with the largest decrease in abundance after migration was 15-lipoxygenase (ALOX15), fold change 0.23 (**Supplementary Table 2**, **Figure 3B**), an enzyme producing pro-resolving lipid mediators shown to inhibit CD4^+^ T cell proliferation (31–33). Of note, the ROS producing enzyme NOX2 (CYBB), was less abundant in migrated compared to incubated neutrophils, fold change 0.64 (**Supplementary Table 2**, **Figure 3B**), in line with the decreased capacity for oxidative burst following migration. Additional proteins possibly involved in the interplay between neutrophils and T cells were significantly decreased, including cofilin-1 (CFL1), dynactin (DCTN1) and dynein (DYNLL2), involved in cell-cell communication *via* neuronal and immune synapses (34–36) (**Supplementary Table 2**, **Figure 3B**).

Next, we performed an enrichment analysis for Gene Ontology Biological Processes on the significantly altered proteins to explore their biological function (**Figure 3B**). In addition to processes involving neutrophil activation and degranulation, processes such as antigen processing and presentation and neuron projection organization (under the biological process of cellular projection organization) were significantly enriched (**Figure 3B**). This indicates that cytoskeletal structure and cell-cell contact is altered upon migration and might be involved in the neutrophil suppression of T cells.

## Discussion

Neutrophils are important communicators in the immune response, with the capacity to both potentiate and suppress activation of the immune system. In this study we demonstrate that neutrophils in synovial fluid of JIA patients can lose the capacity to suppress activated T cells. We used a transwell model of an inflamed joint to unravel the mechanisms behind the loss of neutrophil immunosuppressive capacity and found that migration induced decreased ROS production and alterations in proteins involved in inflammation and cell-cell contact. We hypothesize that inability to suppress T cells at a site of inflammation may contribute to the sustained inflammation and possibly development of local autoimmunity in the joint.

Neutrophils can suppress T cells, but there are controversies about what type of neutrophils that possess this effect. Some studies claim that T cell suppressive effect is restricted to certain neutrophil subsets, including myeloid-derived suppressor cells (MDSC) and neutrophil subpopulations present in different diseases (11, 25, 37, 38), while others argue that normal, healthy neutrophils can inhibit T cell activation and proliferation (7–10). In this study, circulating neutrophils from both healthy controls and children with oligoarticular JIA inhibited T cell proliferation. In contrast, *in vitro* transmigrated neutrophils and neutrophils present in synovial fluid of several of the JIA patients studied were completely non-suppressive. There were no obvious clinical traits that could distinguish the two groups of patients with synovial neutrophils with or without suppressive capacity. One could speculate that the patients might have different immunological pathophysiology, despite being clinically and phenotypically similar. Further research is needed to identify underlying reasons for the dichotomous effect on T cell proliferation.

The phenotype of synovial fluid neutrophils was different between patients with neutrophils of high- and low suppressive capacity. Non-suppressive synovial fluid neutrophils had low levels of the maturation marker CD10, in line with results from a study by Marini et al. where CD10+ neutrophils were immunosuppressive while CD10- neutrophils opposingly were immunostimulatory (25). Neutrophil activation has been described to enhance neutrophil capacity to suppress T cells (6), but in this study there was no significant difference in activation markers CD66b and CD11b on suppressive- compared to non-suppressive neutrophils, suggesting that the absent suppression is not due to lack of neutrophil activation.

The phenotypical differences in surface marker expression between suppressive- and non-suppressive neutrophils in patient synovial fluid was not reflected in the transwell model, suggesting that transmigration and synovial fluid exposure for a short period of time is not enough to fully mimic the phenotype of *in vivo* migrated neutrophils in inflamed joints. CD62L was the only surface marker significantly decreased on *in vitro*-migrated neutrophils compared to non-migrated neutrophils exposed to the same synovial fluid, indicating active migration.

One of the major mechanisms described for neutrophil mediated T cell suppression is the release of ROS (6, 26, 27, 39). In line with this, our transwell-migrated non-suppressive neutrophils had impaired production of ROS and lower levels of NOX2 compared to non-migrated neutrophils. We have previously seen that oligoarticular JIA synovial fluid neutrophils tend to have impaired oxidative burst compared to neutrophils in the circulation (2), and low ROS production might be part of the explanation to why synovial neutrophils from several of the patients in this study are unable to suppress T cell proliferation. However, as ROS scavenging by catalase could only partly neutralize neutrophil-mediated inhibition of T cell proliferation, most likely other mechanisms for T cell suppression are also involved.

The analysis of proteomic alterations induced by migration in our model system identified proteins and processes which might be involved in neutrophil suppression of activated T cells. The proteins with the largest increase and decrease in abundance after migration were clusterin and 15-lipoxygenase respectively. Clusterin, increased upon migration, is involved in multiple immune related processes and has roles in regulating apoptosis and proliferation (30). Clusterin can act immunostimulatory and has been described to induce proliferation and IFNγ production in natural killer cells (40). 15-lipoxygenase, decreased upon migration, produces anti-inflammatory, pro-resolving lipid metabolites which can inhibit CD4^+^ T cell activation and support development of Tregs (32, 33, 41). This increase in immunostimulatory proteins and decrease in anti-inflammatory mediators may contribute to the shift from suppressive to non-suppressive neutrophils upon migration. The proteomic data also indicate that decreased abundance of proteins involved in cell-cell contact and immune synapse regulation might contribute to the loss of suppressive capacity. Indeed, neutrophils have been shown to lose their suppressive capacity on T cells when the two cell types are separated by a membrane with pores too small for migration (6).

The loss of neutrophil suppressive capacity upon migration might be a general phenomenon, not restricted to migration towards inflamed joints. Possibly, neutrophil transendothelial migration towards other tissues and chemotactic stimuli might induce similar neutrophil alterations, which would be interesting to investigate in future studies.

There are some limitations to our study. We could not detect any clinical differences between patients with suppressive- and non-suppressive synovial fluid neutrophils at the time of sampling, but it is possible that there are differences in previous disease- or treatment history between the patient groups which could have contributed to the neutrophil suppressive capacity. In addition, our transwell model, which mimics the non-suppressive synovial neutrophils, could not induce surface marker pattern of synovial fluid neutrophils. This may suggest that the lack of suppressive capacity *in vivo* might be dependent on more complex mechanisms than migration alone. To minimize the risk of inter-individual variations, a single T cell donor could have been used in all experiments. We have used several healthy donors, but within each experiment both neutrophils and T cells were from a single healthy donor.

In conclusion, we demonstrate that synovial fluid neutrophils from several patients with oligoarticular JIA lack capacity to suppress activated T cells, in contrast to circulating neutrophils. We could replicate the loss of suppressive capacity in healthy blood neutrophils in a model of migration over an artificial synovial membrane. Data from the migration model suggest that the loss of suppression is mediated by reduced ROS production and cell-cell interaction, as well as alterations in immunostimulatory and immunoregulatory proteins. We believe that this interplay between the innate immunity (neutrophils) and adaptive immunity (T cells) might contribute to sustained inflammation and development of local autoimmunity in JIA.

## Data Availability Statement

Anonymized datasets used and/or analyzed during the current study are available from the corresponding author on reasonable request. Proteomic data are available at ProteomeXchange (http://www.proteomexchange.org/), accession number PXD029129.

## Ethics Statement

The studies involving human participants were reviewed and approved by Regional Ethical Review Board for Southern Sweden. Written informed consent to participate in this study was provided by the participants’ legal guardian/next of kin.

## Author Contributions

SA-B conceptualized the study, performed experiments and data analysis, interpreted data, and wrote the manuscript. AM and TS performed experiments and reviewed and revised the manuscript. CW and HY performed the LC-MS procedure and reviewed and revised the manuscript. EB and PK collected clinical data and samples as well as reviewed and revised the manuscript. RK conceptualized the study, collected clinical data and samples, interpreted data, and wrote the manuscript. All authors contributed to the article and approved the submitted version.

## Funding

This study was supported by grants from the Swedish Rheumatism Association, Greta and Johan Kock’s Foundation, the Anna-Greta Crafoord Foundation, the Crafoord Foundation, the Swedish Medical Society, Alfred Österlunds Foundation, Magnus Bergvall Foundation, Thelma Zoega’s foundation, King Gustaf V’s 80-year foundation, The Knut and Alice Wallenberg foundation, the Medical Faculty at Lund University and Region Skåne (all to RK). The funders had no role in the concept, design, or interpretation of data.

## Conflict of Interest

The authors declare that the research was conducted in the absence of any commercial or financial relationships that could be construed as a potential conflict of interest.

## Publisher’s Note

All claims expressed in this article are solely those of the authors and do not necessarily represent those of their affiliated organizations, or those of the publisher, the editors and the reviewers. Any product that may be evaluated in this article, or claim that may be made by its manufacturer, is not guaranteed or endorsed by the publisher.
