# Supplementary figures and images for: Neutrophils Lose the Capacity to Suppress T Cell Proliferation Upon Migration Towards Inflamed Joints in Juvenile Idiopathic Arthritis
Source: Front Immunol. 2022 Jan 13;12:795260. doi: 10.3389/fimmu.2021.795260 (PMC8792960; doi:10.3389/fimmu.2021.795260)

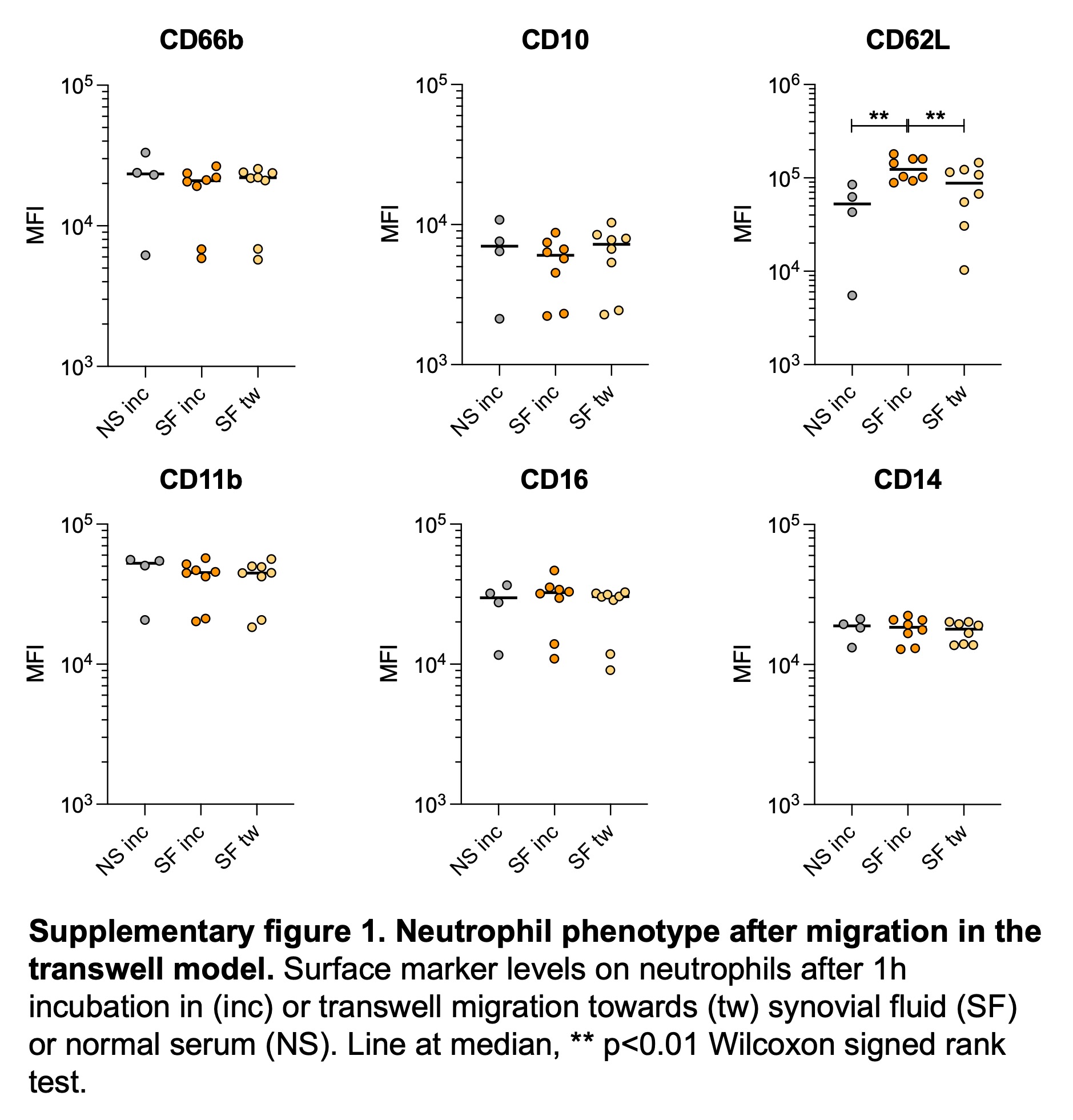

Supplement: Supplementary file 1 [file Image_1.jpeg]
