# Supplementary material for: Neutrophils Lose the Capacity to Suppress T Cell Proliferation Upon Migration Towards Inflamed Joints in Juvenile Idiopathic Arthritis
Source: Front Immunol. 2022 Jan 13;12:795260. doi: 10.3389/fimmu.2021.795260 (PMC8792960; doi:10.3389/fimmu.2021.795260)
Supplement: Supplementary file 2 [file Table_1.docx]

**Supplementary table 1.** Leukocyte count and differential in blood and synovial fluid samples.

| **Patient** | **Fluid** | **Total WBC/ml** | **Neutrophils (%)** | **Lymphocytes (%)** | **Monocytes (%)** | **Eosinophils**  **(%)** |
| --- | --- | --- | --- | --- | --- | --- |
| **1** | Blood | 9,62 | 61,5 | 27,1 | 8 | 2,7 |
|  | SF | 16,83 | 86,9 | 7,6 | 4,8 | 0 |
| **2** | Blood | N/A | N/A | N/A | N/A | N/A |
|  | SF | 0,95 | 27,6 | 49,5 | 21,9 | 0 |
| **3** | Blood | * | * | * | * | * |
|  | SF | 6,45 | 51,1 | 32,6 | 14,7 | 0 |
| **4** | Blood | N/A | N/A | N/A | N/A | N/A |
|  | SF | 19,78 | 57,2 | 25,5 | 12,6 | 0,4 |
| **5** | Blood | 6,89 | 57,2 | 31,1 | 10,2 | 0,6 |
|  | SF | 17,2 | 82,3 | 8,1 | 4,4 | 2,9 |
| **6** | Blood | 6,49 | 62,7 | 26,2 | 8,8 | 1,8 |
|  | SF | 1,19 | 42,3 | 43,1 | 11,4 | 1,1 |
| **7** | Blood | 8,83 | 66,6 | 24,2 | 7,8 | 1,1 |
|  | SF | 17,5 | 72,5 | 20,0 | 6,4 | 0 |
| **8** | Blood | 7,6 | 61,7 | 27,2 | 6,7 | 3,9 |
|  | SF | 2,65 | 32,2 | 50,9 | 11,8 | 0 |
| **9** | Blood | 4,56 | 50,5 | 37,7 | 7 | 3,5 |
|  | SF | 5,77 | 38,6 | 36,5 | 20,8 | 0 |
| **10** | Blood | 4,73 | 68,1 | 22,4 | 7,4 | 1,5 |
|  | SF | 5,28 | 74,1 | 8 | 16,7 | 0 |
| **11** | Blood | 6,11 | 61,6 | 28,8 | 7,4 | 1,5 |
|  | SF | 10,56 | 85,8 | 10,5 | 2,8 | 0 |

WBC – white blood cells, SF – synovial fluid, N/A – no blood sample obtained, * blood sample obtained but cell count and differential data not available
